# Cladribine Induces ATF4 Mediated Apoptosis and Synergizes with SAHA in Diffuse Large B-Cell Lymphoma Cells

**DOI:** 10.7150/ijms.41793

**Published:** 2020-05-30

**Authors:** Linyan Xu, Jun Jiao, Xiaoshen Sun, Wei Sang, Xiang Gao, Pu Yang, Dongmei Yan, Xuguang Song, Cai Sun, Mengdi Liu, Yuanyuan Qin, Yu Tian, Feng Zhu, Lingyu Zeng, Zhenyu Li, Kailin Xu

**Affiliations:** 1 Blood Diseases Institute, Xuzhou Medical University, Xuzhou, Jiangsu, China.; 2 Department of Hematology, the Affiliated Hospital of Xuzhou Medical University, Xuzhou, Jiangsu, China.; 3 Key Laboratory of Bone Marrow Stem Cell, Xuzhou, Jiangsu, China.; 4 Department of Hematology, Luoyang Central Hospital Affiliated to Zhengzhou University, Luoyang, Henan, China.

**Keywords:** cladribine, ATF4, SAHA, apoptosis, DLBCL

## Abstract

Cladribine is a purine nucleoside analog used to treat B-cell chronic lymphocytic leukemia and hairy cell leukemia, also functions as an inhibitor of DNA synthesis to block the repair of the damaged DNA. The therapeutic role of cladribine against diffuse large B-cell lymphoma cells (DLBCL) is still undefined. In the present study, we demonstrated that cladribine inhibited cell proliferation and induced G_1_ phase arrest in human DLBCL cells. Furthermore, we showed that cladribine induced apoptosis by decreasing the expression of c-FLIP_L_ and increasing the expression of DR4 and the cleaved form of caspase8. Cladribine also upregulated the expression of Bax, and downregulated the expression of Mcl-1 and Bcl-2 in a dose-dependent manner. It also activated endoplasmic reticulum (ER) stress, and ATF4 expression was required for cladribine induced apoptosis. Also, we showed that suberoylanilide hydroxamic acid (SAHA) enhanced the pro-apoptotic role of cladribine. Collectively, cladribine activated extrinsic and intrinsic apoptotic signaling pathways via stimulating ER stress signaling pathway and eliciting synergistic effect with SAHA in DLBCL cells.

## Introduction

Diffuse large B-cell lymphoma (DLBCL) is a heterogeneous disease in molecular biology, histomorphology, gene expression, and clinical prognosis, and the incidence rate is increasing annually 1. For most patients, DLBCL is readily curable with immunochemotherapy R-CHOP regimen; however, 30-40% of patients are relapsed, and about 10% of patients are primary refractory 2, 3. The standard second-line of treatment for relapsed and refractory (R/R) DLBCL is autologous stem cell transplantation (ASCT) 2. However, several patients who still cannot be cured succumb to the tumor 4. Nowadays, an important objective is to develop competent and personalized therapeutic strategies.

Cladribine (2-chloro-2′-deoxyadenosine, 2-CdA), a purine analog, is an intermediate for the synthesis of 2-deoxyribonucleotide 5. It is a prodrug and enters the cells through the nucleoside transporters 6. This, in turn, results in the accumulation of active metabolite within the cell, resulting in disruption of cellular metabolism, DNA damage, and subsequent apoptosis 7,8. Cladribine exhibits selective toxicity toward both proliferative and non-proliferative lymphocytes 9. It can also decrease DNA methylation by indirectly inhibiting DNA methyltransferase and consuming methyl donors, thus promoting cell apoptosis 10, 11. Cladribine has anti-lymphoma activity and has been widely used in the treatment of several hematologic malignancies, including hairy cell leukemia (HCL) and chronic lymphocytic leukemia (CLL) and achieved satisfactory results 12-14. Cladribine is also an effective therapeutic agent in the management of mantle cell lymphoma (MCL), follicular lymphoma (FL), and extra nodal B-cell marginal zone lymphoma of the mucosa-associated lymphoid tissue (MALT) 12,15,16. However, the role of cladribine in DLBCL is not well documented.

The combined application of drugs is also an effective strategy for the treatment of cancers 17. The two important epigenetic modifications, methylation and acetylation, play significant roles in controlling gene expression. They have been implicated in aberrant oncogene expression in various cancers 18-20. Recent reports have shown that both DNA methyltransferase and histone deacetylase (HDAC) inhibitors can synergistically cause global alterations in gene expression responsible for cell proliferation, differentiation, and death 21,22. Cladribine can inhibit DNA methylation 10,11, while suberoylanilide hydroxamic acid (SAHA), which is also known as vorinostat, is a powerful HDAC inhibitor that arrests growth and causes apoptosis in many types of cancers 23. Our previous study has shown that SAHA and cladribine synergistically induce apoptosis in natural killer-large granular lymphocyte leukemia (NK-LGLL) 24. Cladribine and SAHA, in combination with gemcitabine and busulfan, also provided synergistic cytotoxicity in lymphoma cell lines 25. Stephen E. Spurgeon showed a phase 1/2 study, which using SAHA, cladribine, and rituximab in relapsed B-cell non-Hodgkin lymphoma (NHL) and previously untreated MCL resulted in a high complete response rate 26. In this study, we also tried to investigate whether combined SAHA and cladribine have a synergistic effect in DLBCL cells.

Apoptosis is recognized as an important programmed cell death; however, the apoptotic program is often decreased in hematologic malignancies. Several anti-cancer therapies and antineoplastic drugs induce apoptosis to eliminate tumor cells; hence, induction of apoptosis is considered one of the effective approaches of antitumor therapy 27-30. Prolonged or severe endoplasmic reticulum (ER) stress impairs the normal functioning of the cells, and could induce cell apoptosis to remove damaged cells 31. The activating transcription factor 4 (ATF4) is a stress-responsive protein that shows elevated expression in ER stress. Several stress signals, namely hypoxia, amino acid deficiency, and oxidative stress, can induce ER stress activation and elevate ATF4 expression 32,33. There is an increased expression of ATF4 in cancer cells, and it promotes apoptosis induced by chemotherapeutic drugs by increasing the expression of DNA damage-inducible transcript 3 (DDIT3) 34,35.

In the present study, we investigated the effect of cladribine on proliferation and cell cycle in DLBCL cells and established the role of cladribine induced apoptosis both in intrinsic and extrinsic pathways. Meanwhile, we testified that ATF4 played an important role in cladribine mediated apoptosis.

## Materials and methods

### Cell culture

The human DLBCL cell lines U2932, OCI-LY10, SUDHL2, WSU-DLCL2, and DB were originally obtained from the American Type Culture Collection (Manassas, VA, USA) and grown in IMDM medium. HEK293T cells were cultured in DMEM medium. All cells were cultured with 10% fetal bovine serum at 37 °C in a humidified atmosphere consisting of 5% CO_2_.

### Antibody and reagents

Compound cladribine was purchased from Sigma-Aldrich (Saint Louis, Missouri, US). The c-FLIP antibody was purchased from EZNO Life Sciences (Farmington, NY, USA). Cyclin D1, Cyclin E, p21, p27, DR4, caspase8, caspase3, PARP, Bax, Mcl-1, Bcl-2, ATF3, CHOP, ATF4, and β-actin antibodies were purchased from Cell Signaling Technology (Danvers, MA, USA).

### Cell viability assay

Cells were seeded in 96-well plates with 2×10^4^ cells per well and treated with cladribine with the indicated concentrations for indicated time. Then Cell Counting Kit-8 (CCK-8) (Dojindo Laboratories, Kumamoto, Japan) was used to measure cell viability according to the manufacturer's instruction.

### Western blot

Human DLBCL cells were harvested, lysed, and detected protein concentrations by BCA assay. Then 50 µg protein samples were separated by SDS-PAGE electrophoresis, transferred to PVDF membranes, blocked with 5% skim milk and incubated with primary antibodies at 4 °C overnight. After washing the membranes and incubating with horseradish peroxidase-conjugated secondary antibodies, bounded antibodies were detected by enhanced chemiluminescence (ECL) kit (BD Biosciences, San Jose, CA, USA), and pictures were captured using GE Image Quant LAS4000 mini.

### Apoptosis assay

Apoptosis was detected by Annexin V-PE/7-AAD apoptosis detection kit purchased from BD Biosciences following the manufacture's protocol. Briefly, the cells were seeded in 6-well plates with 2×10^6^ cells per well and treated with cladribine for 24 h. Then harvest the cells and resuspend with binding buffer containing 5 µL Annexin V-PE and 5 µL 7-AAD (BD Biosciences, San Jose, CA, USA). At the same time, we conducted three controls used to set up compensation and quadrants: unstained cells, cells only stained with 5 µL Annexin V-PE, cells only stained with 5 µL 7-AAD. Samples were incubated in the dark for 15 minutes and detected by flow cytometer within 1 h. All data were analyzed using FlowJo software.

### Cell cycle analysis

Cell cycle analysis was detected by flow cytometry. Cells were seeded in 6-well plates with 2×10^6^ cells per well and treated with cladribine for 24 h. Then harvest the cells and fix with 70% ice-cold ethanol at -20 °C. The next day, the cells were washed and resuspended with PBS. Meanwhile, stain the cells with propidium iodide (contain RNase A: 50 µg/mL) for 30 min protection from light and then detect the cells using a flow cytometer. Measure the forward scatter (FS), side scatter (SS) to identify cells and use pulse area, pulse width to exclude cell debris, clumps, and doublets. Single cells were acquired with the maximum emission of PI less than 605 nm and analyzed the data by ModFit LT 3.3 software (Verity Software House Inc., Topsham, ME, USA).

### RNA extraction and real-time qPCR

The cells were collected, and RNA was extracted with TRIzol^™^ reagent (Life Technologies, Gaithersburg, MD, USA) according to the manufacturer's instruction. The concentration was measured at 260 nm, and RNA was used to synthesize to cDNA using M-MLV reverse transcriptase (Life Technologies, Gaithersburg, MD, USA). Then q-PCR was performed according to the manufacturer's instruction using SYBR^®^ Green Supermix (Roche, Indianapolis, IN, USA). Sequences of primers for RT-qPCR genes are listed in Table 1. The PCR amplification was conducted using the following procedure: 94 °C for 2 min, followed by 35 cycles of 94 °C for 30 s, 60 °C for 30 s, 72 °C for 35 s, and a final extension at 72 °C for 2 min. The threshold cycle number (Ct) was calculated, and the relative mRNA expression of target genes was calculated by 2^-ΔΔCt^ method.

### ShRNA oligonucleotide design and construction of stable cell lines

The ATF4 shRNA oligonucleotide sequences were designed as 5'-GCCTAGGTCTCTTAGATGATT-3' (1), 5'-GCCAAGCACTTCAAACCTCAT-3' (2). After cloning shRNA sequences into a pLVshRNA-eGFP vector, the desired plasmids were transfected into HEK-293T cells accompanied by packaging plasmid psPAX2 and envelope plasmid pMD2.G using transfection reagent Lipofectamine 2000 (Invitrogen, Carlsbad, CA, USA). Collect the lentivirus supernatant and concentrate the lentivirus for infection into DLBCL cells. The infected cells were then treated with puromycin for screening stable cell lines. Finally, the screened stable cells were identified by flow cytometry and western blot.

### Statistical analysis

Data were shown as means ± standard deviation (SD). Two-tailed unpaired Student's t-test was used to analyze the statistical significance between two treatment groups. One-way ANOVAs was used to analyze the statistical significance between multiple treatment groups. Statistical significance was established as *P<0.05, **P<0.01, ***P<0.001. All statistical analyses were performed using the GraphPad Prism software ver. 6.0 (GraphPad Software Inc., La Jolla, CA, USA).

## Results

### Cladribine inhibits human DLBCL cells proliferation

**Figure 1:**
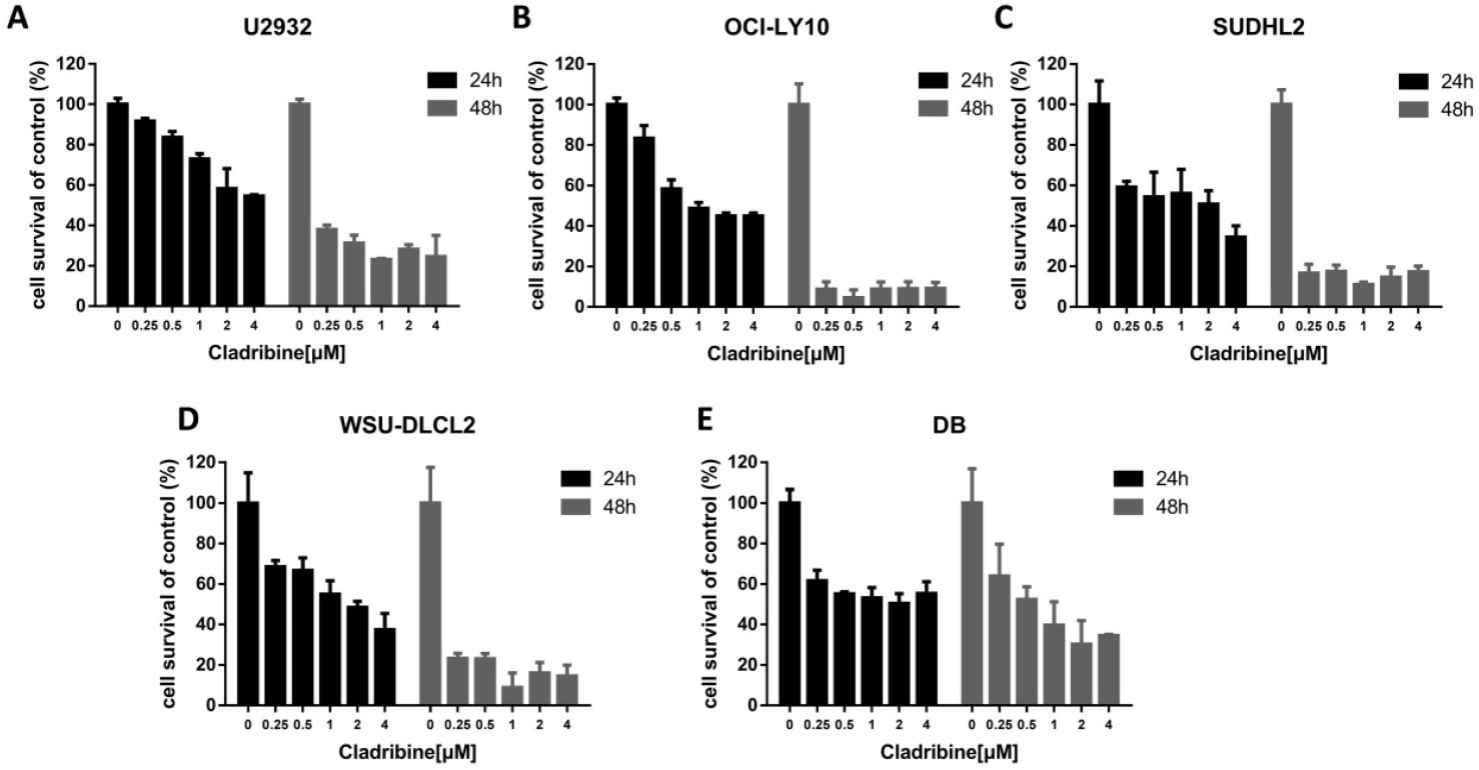
** Cladribine inhibits human DLBCL cells proliferation. A-E.** U2932 (A), OCI-LY10 (B), SUDHL2 (C), WSU-DLCL2 (D) and DB (E) cells were incubated with the indicated concentrations of cladribine for 24 h or 48 h, then CCK-8 assay was performed to detect the viability. Error bars, mean ± SD.

To determine the effect of cladribine on the growth of DLBCL cells, CCK-8 assay was performed to detect the viability. As shown in Figure 1A-E, cladribine exhibited notable suppression of cell proliferation in five DLBCL cells. Concurrently, it was observed that the inhibitory effect was more profound with the time elapse.

### Cladribine induces G_1_ phase arrest in human DLBCL cells

**Figure 2:**
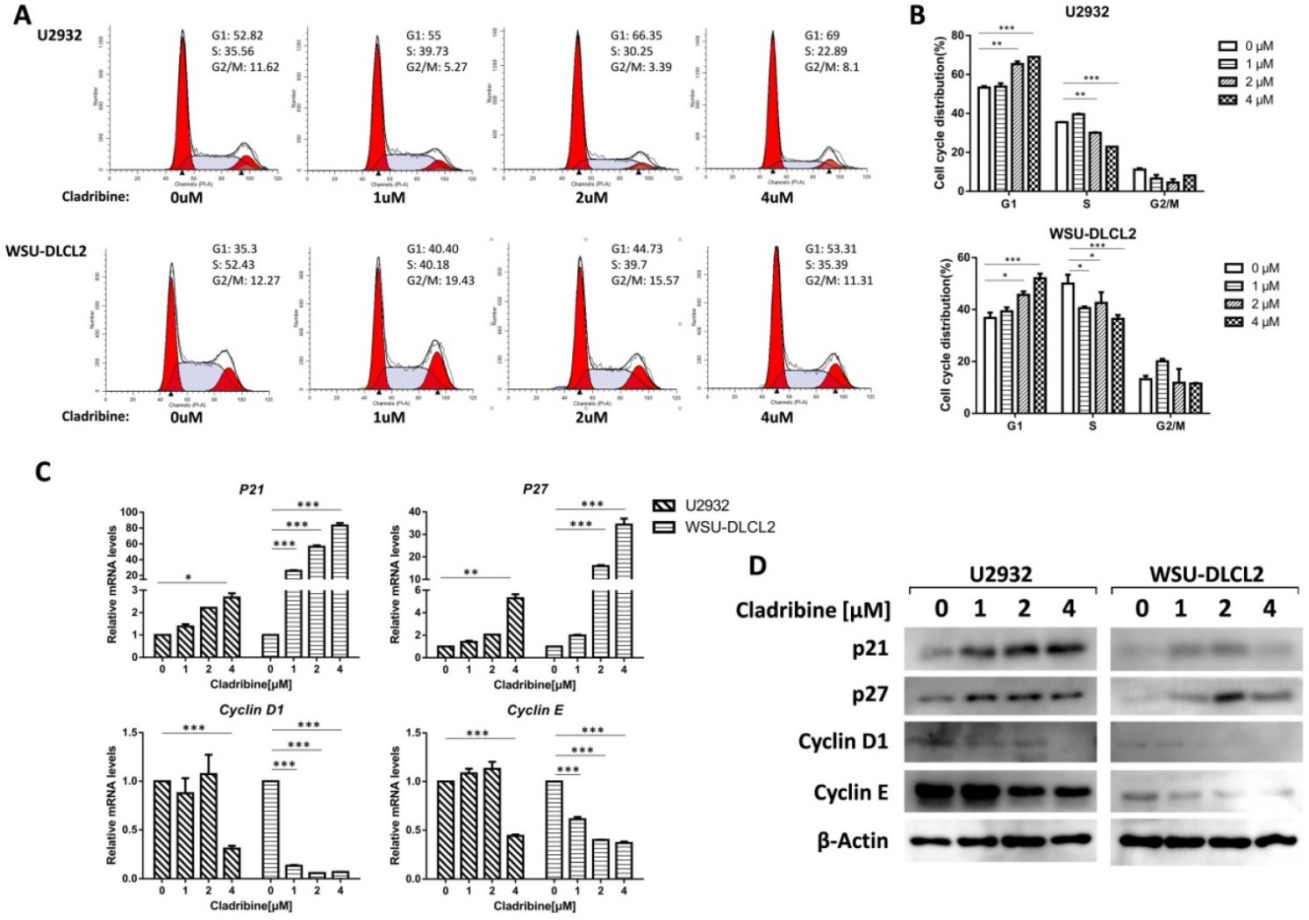
** Cladribine induces G_1_ phase arrest in human DLBCL cells. A.** U2932 and WSU-DLCL2 cells were incubated with the indicated concentrations of cladribine for 24 h. Then cells were harvested and prepared for cell cycle analysis. **B.** Percentages of the subpopulation of cells at different cell cycle phases were determined from three independent experiments.** C.** U2932 and WSU-DLCL2 cells were incubated with the indicated concentrations of cladribine for 24 h. The expressions of *Cyclin D1*, *Cyclin E*, *P21,* and *P27* mRNA were assessed by real-time PCR. Error bars, mean ± SD. *P < 0.05; **P < 0.01; ***P < 0.001. **D.** U2932 and WSU-DLCL2 cells were incubated with the indicated concentrations of cladribine for 24 h. Then whole cells were harvested and subjected to western blot using Cyclin D1, Cyclin E, p21, and p27 antibodies.

To further study the underlying mechanism of the inhibition of DLBCL cell proliferation by cladribine, flow cytometry analysis was carried out to investigate the cell cycle distribution after treating the cells with cladribine. The cells in the G_1_ phase were increased in a dose-dependent fashion both in U2932 and WSU-DLCL2 cells, while the population in the S phase was decreased from 35.56% to 22.89% in U2932 cells and from 52.43% to 35.39% in WSU-DLCL2 cells (Figure 2A and 2B). Moreover, when the mechanism of G_1_ phase arrest was studied, the mRNA levels of *Cyclin D1* and *Cyclin E* were found to decrease, while those of *P21* and *P27* were increased (Figure 2C). Moreover, western blot showed the expressions of Cyclin D1 and Cyclin E were decreased, while there were elevated expressions of p21 and p27 in U2932 and WSU-DLCL2 cells (Figure 2D). Taken together, these results indicate that cladribine causes G_1_ phase arrest via decreasing the expressions of Cyclin D1 and Cyclin E, and increasing the expressions of p21 and p27 in DLBCL cells.

### Cladribine induces apoptosis and activates extrinsic and intrinsic signaling pathways in human DLBCL cells

**Figure 3:**
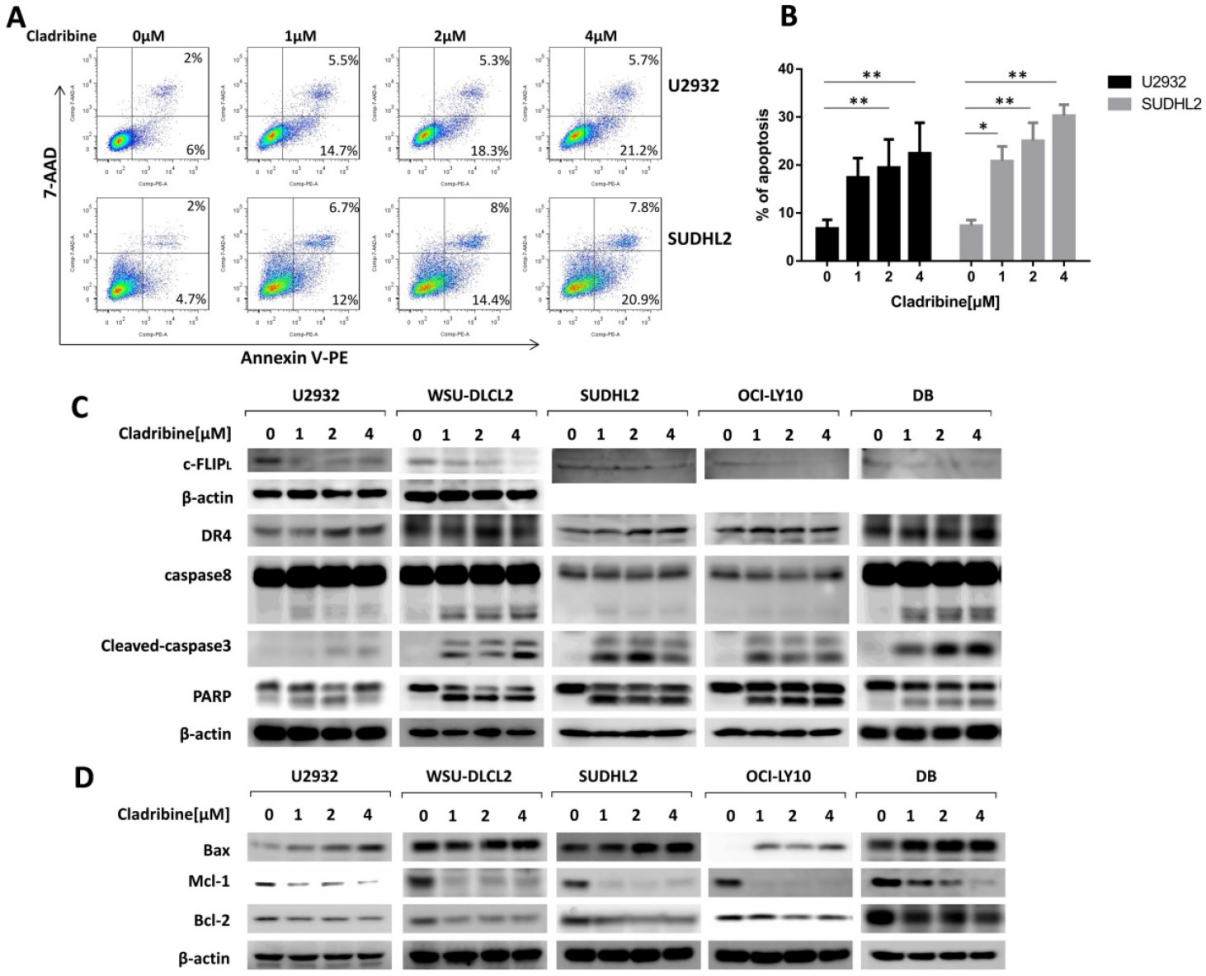
** Cladribine induces apoptosis and activates exogenous and endogenous apoptotic signaling pathways in human DLBCL cells. A.** U2932 and SUDHL2 cells were incubated with the indicated concentrations of cladribine for 24 h, and then cells were harvested and subsequently stained with Annexin-V-PE and 7-AAD and analyzed by flow cytometry for apoptosis. **B.** Percentages of apoptotic cells were determined from three independent experiments. Error bars, mean ± SD. *P < 0.05; **P < 0.01. **C and D.** U2932, WSU-DLCL2, SUDHL2, OCI-LY10, and DB cells were incubated with the indicated concentrations of cladribine for 24 h. Then whole cells were harvested and subjected to western blot using c-FLIP, DR4, caspase8, caspase3, PARP (C) and Bax, Mcl-1, Bcl-2 (D) antibodies.

Furthermore, we performed a flow cytometric assay to elucidate the apoptotic effect and found that cladribine treatment induced apoptosis of U2932 and SUDHL2, and its percentage significantly increased with an increase in concentration (Figure 3A and 3B). The apoptotic signaling pathway was further activated. As shown by western blotting, the level of death receptor DR4 was upregulated in U2932, OCI-LY10, SUDHL2, WSU-DLCL2, and DB cells (Figure 3C). The expression of anti-apoptotic protein c-FLIP was decreased, and the cleavage of caspase8 was elevated in these cells (Figure 3C). Moreover, cladribine treatment increased the cleaved forms of caspase3 and PARP, indicating that it induces the extrinsic apoptotic pathway. Furthermore, we examined that cladribine raised the expression of pro-apoptotic protein Bax, and reduced the expression of anti-apoptotic proteins Mcl-1 and Bcl-2 in a dose-dependent manner (Figure 3D), suggesting the role of cladribine in inducing intrinsic apoptotic pathway. Taken together, these results indicate cladribine induces apoptosis and activates extrinsic and intrinsic signaling pathways in human DLBCL cells.

### Cladribine activates endoplasmic reticulum stress

**Figure 4:**
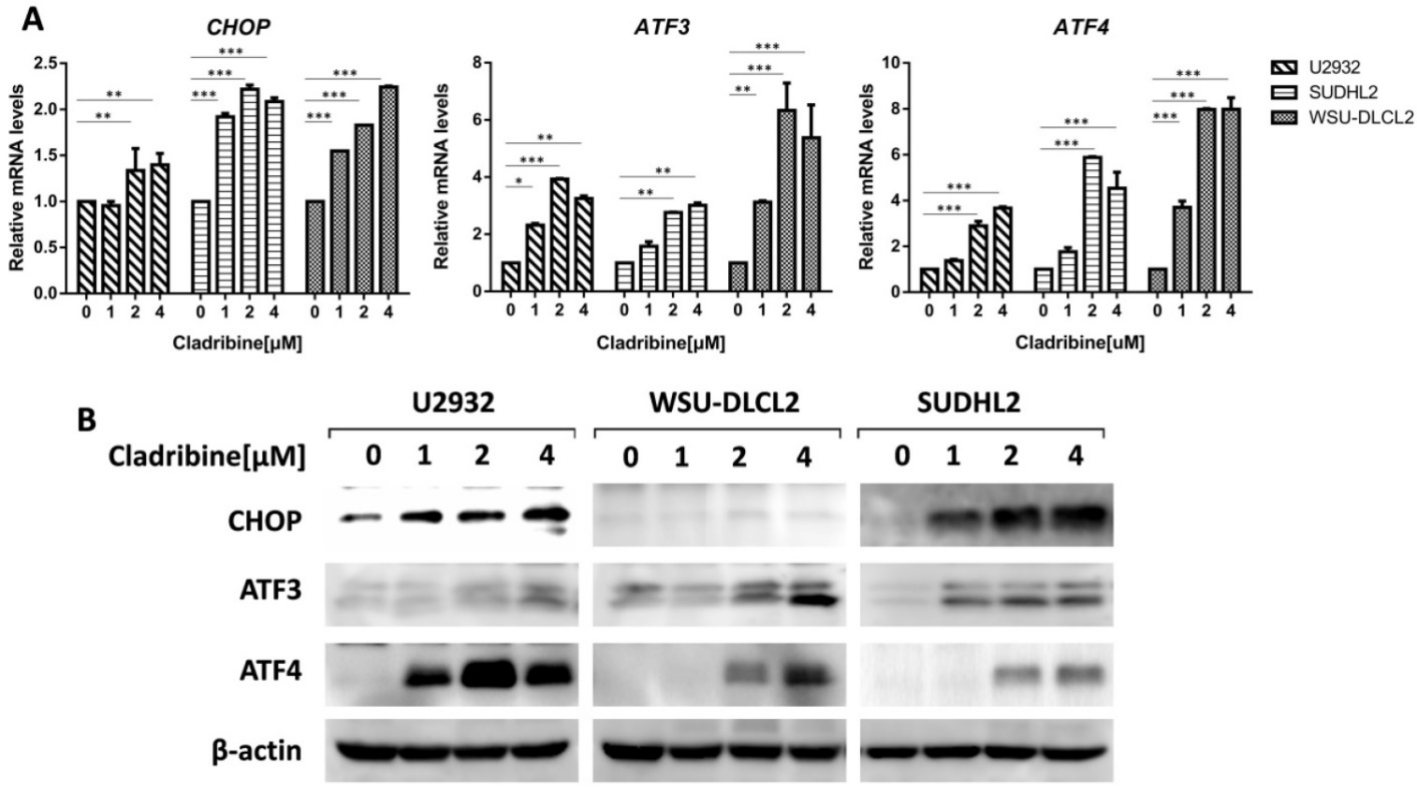
** Cladribine activates ER stress. A-B.** U2932, SUDHL2 and WSU-DLCL2 cells were incubated with the indicated concentrations of cladribine for 24 h, and then whole cells were harvested and subjected to real-time PCR assay (A) or western blot analysis using ATF3, CHOP, and ATF4 antibodies (B). Error bars, mean ± SD. *P < 0.05; **P < 0.01; ***P < 0.001.

To elucidate the mechanism of cladribine-induced apoptosis in DLBCL cells, we examined the mRNA levels of *CHOP*, *ATF3,* and *ATF4*, which were considered as important markers of ER stress and found that their expressions were enhanced in a dose-dependent fashion (Figure 4A). Moreover, we confirmed that their protein levels were also increased (Figure 4B). Collectively, these results indicate that cladribine activates ER stress.

### ATF4 expression is required for cladribine induced apoptosis

**Figure 5:**
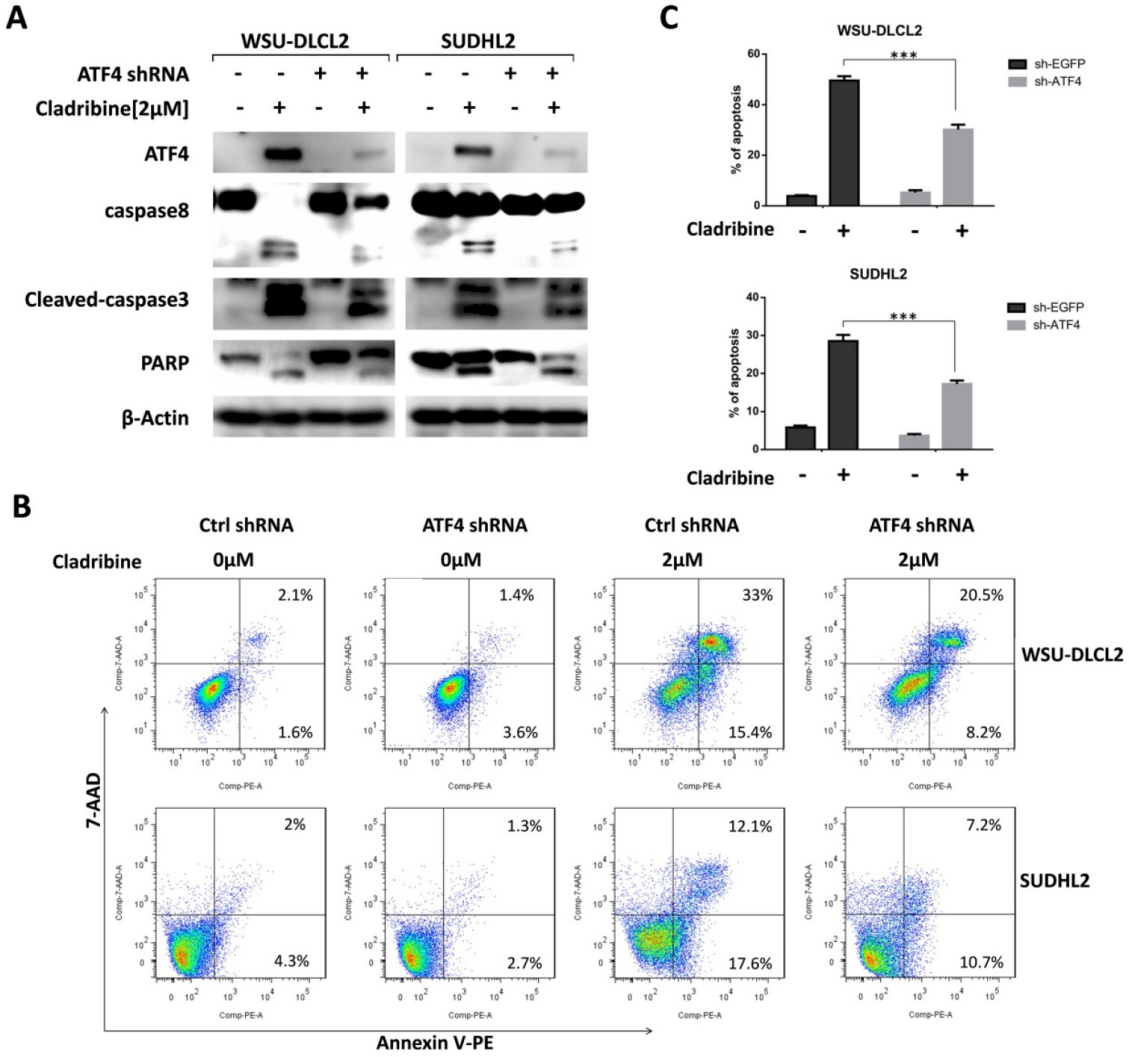
** ATF4 expression is required for cladribine-induced apoptosis. A.** WSU-DLCL2 and SUDHL2 cells were transduced with ATF4-shRNA and control-shRNA lentivirus and constructed stable knockdown cell lines. Then cells were incubated with cladribine for 24 h. Whole cells were harvested and subjected to western blot analysis using ATF4, caspase8, caspase3, and PAPR antibodies. **B and C.** WSU-DLCL2 and SUDHL2 stable cell lines with ATF4 knockdown were incubated with cladribine for 24 h, and then cells were detected with Annexin V/7-AAD by flow cytometry (B), and the percentages of cell apoptosis were determined from three independent experiments (C). Error bars, mean ± SD. ***P < 0.001.

We then focused on the role of ATF4 by designing ATF4-shRNA to inhibit ATF4 expression. The inhibition of ATF4 up-regulation decreased the cleavage of caspase8, caspase3, and PARP in WSU-DLCL2 and SUDHL2 cells (Figure 5A). Consistently, the percentage of apoptotic cells in control shRNA-transfected cells was 48.4%, whereas it was only 28.7% in ATF4 shRNA-transfected WSU-DLCL2 when cells were exposed to cladribine. In SUDHL2 cells, the corresponding proportion was decreased from 29.7% to 17.9% (Figure 5B and 5C) indicating ATF4 contributes to cladribine induced apoptosis.

### SAHA enhances the pro-apoptotic role of cladribine

**Figure 6:**
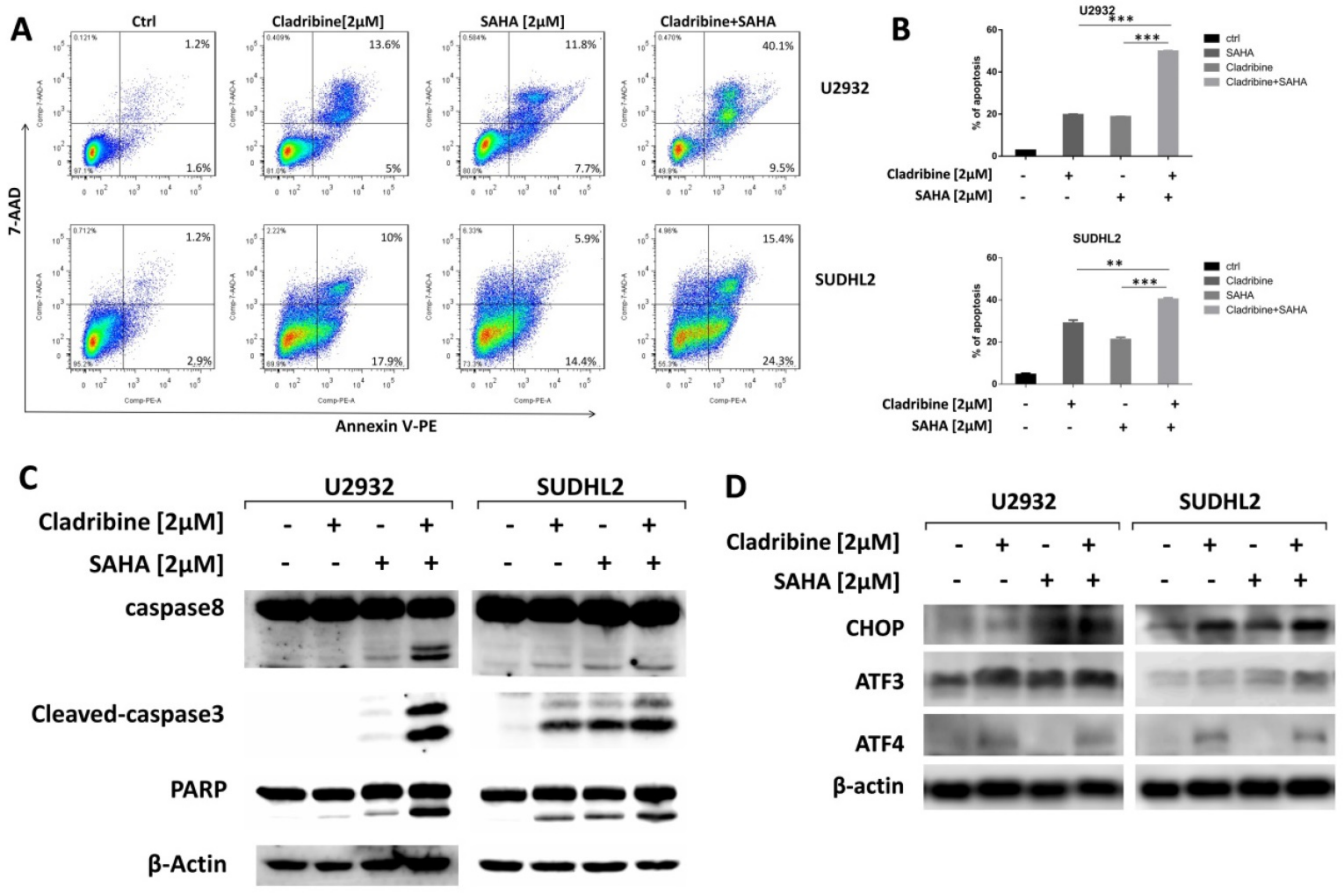
** SAHA enhanced the pro-apoptotic role of cladribine. A and B.** U2932 and SUDHL2 cells were treated with cladribine and SAHA for 24 h. Then the whole cells were harvested and detected with Annexin V/7-AAD by flow cytometry (A), and percentages of cell apoptosis were determined from three independent experiments (B). Error bars, mean ± SD. **P < 0.01; ***P < 0.001. **C and D.** U2932 and SUDHL2 cells were treated with cladribine and SAHA for 24 h. Then the whole cells were harvested and subjected to western blot analysis using caspase8, caspase3, PAPR antibodies (C) and CHOP, ATF3, ATF4 antibodies (D).

To explore whether SAHA enhances the pro-apoptotic effect of cladribine in DLBCL cells, we treated U2932 and SUDHL2 cells with cladribine alone or in combination with SAHA. When cells were treated with a combination of cladribine and SAHA, an increase in apoptosis was detected using Annexin V staining-flow cytometry (Figures 6A and 6B). We confirmed the enhanced cleavage of caspase8, caspase3, and PARP using western blot test in the combination group compared to cladribine or SAHA alone (Figure 6C). In addition, we discovered that SAHA cannot alter the expression of ATF4, and combination of cladribine and SAHA also did not affect the expression of ATF4 (Figure 6D). SAHA slightly increased the expression of CHOP, ATF3, and combination of cladribine and SAHA also increased CHOP and ATF3 expression mildly (Figure 6D). Collectively, these results indicate that SAHA enhanced cladribine-mediated apoptosis, and partially enhanced ER stress.

## Discussion

The prognosis of DLBCL has largely improved with the application of monoclonal anti-CD20 antibody rituximab; however, there is still a group of patients who cannot benefit from the standard treatment. So developing efficacious or specific therapeutic strategies continues to be an important objective 2-4. Cladribine is a purine analog, which has been used in the treatment of B and T cell lymphoid malignancies, including multiple sclerosis, HCL, and CLL 5. Meanwhile, cladribine also has an antitumor role in MCL, FL, and MALT 12-16. In the study, we showed that cladribine inhibited cell proliferation and induced G_1_ phase arrest in human DLBCL cells. Furthermore, we showed that cladribine induced apoptosis and activated ER stress, and ATF4 expression was required for cladribine induced apoptosis. Also, we showed that SAHA enhanced the pro-apoptotic role of cladribine.

In the present study, we showed that cladribine induced apoptosis by decreasing the expression of c-FLIP and increasing the expression of DR4 and the cleaved form of caspase8, indicating that cladribine induced extrinsic apoptotic pathway. It is reported that cladribine induces human leukemia cell line apoptosis through the Fas/Fas ligand pathway 36, our results further established the role of cladribine in inducing extrinsic apoptotic pathway. Furthermore, the expressions of anti-apoptotic proteins Mcl-1 and Bcl-2 were decreased and the expression of pro-apoptotic protein Bax was increased, suggesting that cladribine also activated the intrinsic apoptotic pathway in DLBCL cells. These results were consistent with the earlier studies that cladribine induces apoptosis via activation of caspases 3, 6, 8, and 9 and decreasing the level of the anti-apoptotic protein Mcl-1 37,38. Also, studies have shown cladribine reduces the mitochondrial transmembrane potential, releases of cytochrome C and translocates AIF from mitochondria to the nucleus and causes chromatin condensation 37,39. However, cladribine cannot change Bax and Bcl-2 expression in B-CLL cells in patients who express high levels of Bcl-2, Bax, and Bak proteins 37. The study by V. Singh revealed a contrary view that cladribine induced caspase-independent apoptosis 39,40, so it induces apoptosis via various patterns that may be dependent on different cell types and gene expression. Whether cladribine would induce caspase-independent apoptosis in DLBCL cells remains to be further explored.

The mechanism of cladribine induced apoptosis has been extensively studied. It has been reported that cladribine activates the tumor suppressor p53, which further activates the intrinsic apoptotic pathway 37,41. The intracellular calcium ion concentration, p38 mitogen-activated protein kinase, extracellular signal-regulated kinases 1 and 2 also probably play roles in the induction of apoptosis by cladribine 42,43. In our study, we observed that cladribine elevated CHOP, ATF3, and ATF4 expression, which are important components of ER stress, indicating cladribine activated ER stress. Severe ER stress could induce apoptosis 44,45, and ATF4 promotes apoptosis induced by chemotherapeutic drugs such as bortezomib, cisplatin, and arsenic trioxide 34,46,47. So we evaluated whether ER stress influenced cladribine induced apoptosis. We mainly focused on the important component ATF4 and found that the inhibition of ATF4 upregulation decreased the cleavage of caspases8, 3, and PARP induced by cladribine. Meanwhile, the percentage of apoptotic cells was also decreased in ATF4 shRNA-transfected cells when exposed to cladribine, suggesting that ATF4 participated in cladribine-induced apoptosis. Consistently, Swetlana Mactier reported ER stress pathway is involvement in cladribine induced apoptosis in Raji cells 48.

The combination therapy is gradually becoming a preferable practice in DLBCL treatment. The combination of different drugs might improve the prognosis of patients by suppressing cell proliferation, enhancing cell apoptosis, and surmounting drug resistance 49-51. It has been found that combination of cladribine with other chemotherapy drugs shows good synergistic effects. Cladribine, in combination with bendamustine or STAT3 inhibitor, exhibited inhibitory activity in multiple myeloma cells 52,53. A combination of cladribine with anthracyclines also induced apoptosis in lymphocytes in patients with B-cell chronic lymphocytic leukemia 54. Our previous study also showed that SAHA and cladribine synergistically induced apoptosis in NK-LGL leukemia 24. In the present study, we also investigated the combined synergistic effect of SAHA with cladribine in inducing apoptosis in DLBCL cells. The expressions of CHOP and ATF3 were slightly elevated with a combination of SAHA with cladribine, but the expression of ATF4 was not changed, so the detailed mechanism of the synergistic role needs to be further explored.

In summary, we showed that cladribine inhibited cell proliferation and induced G_1_ phase arrest and apoptosis in human DLBCL cells. We further demonstrated that cladribine activated ER stress, and ATF4 expression was required for cladribine induced apoptosis. Moreover, we showed that SAHA enhanced the pro-apoptotic role of cladribine and slightly enhanced ER stress. Collectively, cladribine activated extrinsic and intrinsic apoptotic signaling pathways by stimulating ATF4 expression and eliciting a synergistic effect with SAHA in DLBCL cells.

## Figures and Tables

**Table 1:** Sequences of primers for RT-qPCR

Name	Sequences
Cyclin D1	5′-GCCCGAGGAGCTGCTGCAAA -3′(forward) 5′-GCAACGAAGGTCTGCGCGTG -3′(reverse)
Cyclin E	5′-TTCTTGAGCAACACCCTCTTCTGCAGCC -3′(forward) 5′-TCGCCATATACCGGTCAAAGAAATCTTGTGCC -3′(reverse)
P21	5′-TGAGCCGCGACTGTGATG-3′(forward) 5′-GTCTCGGTGACAAAGTCGAAGTT-3′(reverse)
P27	5′- TGCAACCGACGATTCTTCTACTCAA-3′(forward) 5′- CAAGCAGTGATGTATCTGATAAACAAGGA -3′(reverse)
ATF4	5′-GCTAAGGCGGGCTCCTCCGA- 3′(forward) 5′-ACCCAACAGGGCATCCAAGTCG-3′(reverse)
ATF3	5′-TGATGCTTCAACACCCAGGCC-3′(forward) 5′-AGGGGACGATGGCAGAAGCA-3′(reverse)
CHOP	5′-CATCACCACACCTGAAAGCA -3′(forward) 5′-TCAGCTGCCATCTCTGCA-3′(reverse)
β-Actin	5′-CTCCATCCTGGCCTCGCTGT -3′(forward) 5′-GCTGTCACCTTCACCGTTCC- 3′(reverse)

## Abbreviations

2-CdA: 2-chloro-2′-deoxyadenosine
ASCT: autologous stem cell transplantation
ATF: activating transcription factor
CCK-8: cell counting kit-8
CHOP: C/EBP homologous protein
CLL: chronic lymphocytic leukemia
DDIT3: DNA damage-inducible transcript 3
DLBCL: diffuse large B-cell lymphoma
DR: death receptor
ECL: enhanced chemiluminescence
ER: endoplasmic reticulum
FS: forward scatter
FL: follicular lymphoma
HCL: hairy cell leukemia
HDAC: histone deacetylase
MALT: mucosa-associated lymphoid tissue
MCL: mantle cell lymphoma
NHL: non-Hodgkin lymphoma
NK-LGLL: natural killer-large granular lymphocyte leukemia
R-CHOP: rituximab, cyclophosphamide, doxorubicin, vincristine, and prednisone
R/R: relapsed and refractory
RT-qPCR: real-time quantitative polymerase chain reaction
SAHA: suberoylanilide hydroxamic acid
SD: standard deviation
SS: side scatter
